# Bioengineered T-cell therapies for precision immunotherapy in renal transplantation, autoimmune relapse, and sensitization

**DOI:** 10.1093/ckj/sfaf248

**Published:** 2025-08-08

**Authors:** Amir Muhammad, Hanwei Huang, Rong Tang, Yingli Zhang, Yuxi Xiao, Qiongjing Yuan, Xiangcheng Xiao

**Affiliations:** Xiangya Hospital Central South University, Department of Nephrology, Changsha, Hunan, China; Xiangya Hospital Central South University, Department of Nephrology, Changsha, Hunan, China; Organ Fibrosis Key Lab of Hunan province, Central South University, Changsha, Hunan, China; Xiangya Hospital Central South University, Department of Nephrology, Changsha, Hunan, China; The Third Hospital of Changsha, Nephrology, Changsha, Hunan, China; Xiangya School of Medicine, Central South University, Changsha, Hunan, China; Xiangya Hospital Central South University, Department of Nephrology, Changsha, Hunan, China; Organ Fibrosis Key Lab of Hunan province, Central South University, Changsha, Hunan, China; National International Collaborative Research Center for Medical Metabolomices, Changsha, Hunan, China; Xiangya Hospital Central South University, Department of Nephrology, Changsha, Hunan, China

**Keywords:** autoimmune relapse, chimeric antigen receptor, end-stage renal failure, prior sensitization, renal transplantation

## Abstract

Patients with end-stage renal disease (ESRD) undergoing dialysis experience higher mortality rates, increased healthcare costs, and reduced quality of life compared to those receiving allogeneic renal transplantation (RT). Although RT provides proven survival and functional benefits, long-term graft outcomes remain limited by the ongoing risk of immune-mediated rejection—even in cases with donor–recipient histocompatibility, except for rare syngeneic (identical twin) transplants. Additionally, the recurrence of primary autoimmune glomerular diseases, which commonly contribute to ESRD pathogenesis, continues to pose a clinical challenge for maintaining stable allograft function and optimizing long-term outcomes. While immunosuppressive therapy remains crucial to the prevention of allograft rejection and autoimmune relapse, its use is frequently associated with clinically significant complications, including nephrotoxicity, opportunistic infections, new-onset diabetes after transplantation, and an elevated risk of malignancy. Chimeric antigen receptor (CAR) regulatory T cells (Tregs) are emerging as a potential alternative to polyclonal or antigen-specific Tregs, offering advantages such as reduced cell dosing requirements and human leukocyte antigens (HLA)-independence. These features may help overcome limitations posed by HLA allele variability in genetically diverse populations. However, the concurrent use of immunosuppressive agents to prevent autoimmune relapse—particularly in conditions such as focal segmental glomerulosclerosis (FSGS), lupus nephritis (LN), and IgA nephropathy (IgAN)—poses challenges to the therapeutic efficacy and safety profile of CAR-Tregs. Furthermore, CAR-Tregs have demonstrated potential in attenuating anti-HLA-A2 IgG donor-specific antibodies (DSAs) in naïve preclinical models, their efficacy in sensitized recipients remains insufficiently established. This review explores the challenges and therapeutic potential of dual CARs and bicistronic CARs in Tregs, alongside chimeric HLA antibody receptor (CHAR) T-cell strategies, to concurrently address RT rejection and autoimmune relapse through CAR-Tregs, and allo-sensitization through CHAR-T cells. Emphasis is placed on optimizing CAR construct design, selecting appropriate target antigens, expanding eligibility criteria, and integrating CAR-Treg therapy with CHAR-T-cell-based desensitization protocols. These approaches collectively aim to improve transplant outcomes, reduce relapse rates, minimize reliance on conventional immunosuppressants, and ensure the safety and efficacy of CAR-Treg and CHAR-T cell therapies.

## INTRODUCTION

Regulatory T cells (Tregs), characterized by CD25^high^ and CD127^low^ expression in humans, are essential for maintaining immune homeostasis by modulating immune responses, preventing autoimmunity, and mitigating inflammatory damage [1]. Advances in cellular immunotherapies have shifted the approach from non-specific immunosuppression to precise immune modulation, as illustrated by chimeric antigen receptor (CAR) T cells in oncology [2]. Building on this platform, CAR-Tregs are bioengineered to recognize specific antigens, enabling antigen-specific immune regulation. While they have demonstrated efficacy in preclinical models targeting human leukocyte antigens (HLA)-restricted antigens, the modularity of CAR design allows redirection toward non-HLA targets, potentially addressing limitations related to HLA compatibility [3]. This flexibility is particularly advantageous given the variability of immunodominant HLA alleles across individuals, which often limits the applicability of polyclonal or conventional antigen-specific Treg therapies [3, 6].

In preclinical models, CAR-Tregs have demonstrated the ability to selectively suppress immune responses against transplanted kidneys, leading to reduced rejection and prolonged graft survival, even at lower cell doses [3–5]. The enhanced precision and functional efficacy of engineered CAR-Tregs stem from their antigen specificity, which overcomes key limitations of both polyclonal and conventional antigen-specific Tregs [3, 7]. In murine models, antigen-specific CAR-Tregs significantly improved graft survival relative to polyclonal or non-specific Tregs [8]. Similarly, in a human skin xenograft model, HLA-A2-specific CAR-Tregs conferred superior protection to HLA-A2⁺ allografts [8]. In contrast, polyclonal Tregs require high cell doses and pose a risk of broad immunosuppression, while traditional antigen-specific Tregs are limited by alloantigen diversity and HLA polymorphism, constraining their clinical scalability [3, 7].

Autoimmune glomerular diseases, such as focal segmental glomerulosclerosis (FSGS), lupus nephritis (LN), IgA nephropathy (IgAN), and ANCA-associated vasculitis (AAV), are significant contributors to end-stage renal disease (ESRD). Following renal transplantation (RT), these conditions remain at risk of recurrence, particularly in the absence of adequate post-transplant immunomodulation [9–13]. Current clinical guidelines recommend sustained immunosuppressive therapy to mitigate both graft rejection and disease relapse, with agents such as rituximab and plasmapheresis used in selected high-risk settings. While immunosuppressive agents are critical in mitigating autoimmune relapse following RT, their concurrent use during CAR-Treg therapy aimed at preventing renal graft rejection may compromise the safety, efficacy, and functional stability of CAR-Tregs [14]. One such concern is the potential pharmacological interaction between standard immunosuppressive agents and molecular components of the CAR-Treg safety switch. For instance, tacrolimus—a calcineurin inhibitor commonly used post-transplantation—binds to FK506-binding protein (FKBP), which is also required for the activation of inducible caspase 9 (iCasp9), a suicide gene incorporated into some CAR-Treg constructs for controlled cell elimination [7]. By competing for FKBP binding, tacrolimus may lead to unwanted activation of iCasp9 in CAR-Tregs, resulting in their elimination and potentially compromising the reliability of this safety mechanism [15, 16]. In addition, the established toxicities of long-term immunosuppression—including nephrotoxicity, infection, and metabolic complications—further complicate relapse prevention in these patients [17]. Current clinical trials, such as NCT04817774 (STEADFAST Trial), along with previous studies like the STEADFAST protocol for living-donor renal transplantation, have not adequately addressed the issue of relapse management in this complex scenario (Table 1) [18]. Notably, these trials exclude patients on systemic immunosuppressive agents, those at higher risk of relapse, and patients with DSAs or sensitization—common among ESRD patients—thereby limiting the applicability and efficacy of CAR-Tregs.

**Table 1:** Study protocol and preclinical studies demonstrating CAR-Tregs post-transplantation and autoimmune diseases.

Studies	Species	Design	CAR-Tregs features	Target antigen	Outcome	Ref.
STEADFAST study protocol; Living-Donor Renal Transplantation	Human beings	Allogeneic transplant of islets from HLA-A02-positive donors into HLA-A02-negative recipients.	CAR-engineered from CD4^+^/CD45RA^+^/CD25^+^/CD127^low/neg^ + naïve Tregs	HLA-A*02	Study protocol; results not yet published.	[18]
Heart Transplantation	Mice	Allogeneic heart transplant from ABO-incompatible donor mice to NSG recipient mice.	CAR-engineered CD62L^+^CD4^+^CD25^+^FoxP3 Tregs	C4d	Improved ABO-incompatible heart allograft survival by suppressing ABMR.	[38]
Islet Transplantation	Cynomolgus macaques	Allogeneic islet transplant from Bw6 + Cynomolgus macaques to diabetic Cynomolgus macaques.	CAR-engineered CD62L^+^CD4^+^CD25^+^FoxP3 Tregs	Bw6 alloantigen	CAR-Tregs trafficked to graft site and maintained a stable suppressive phenotype.	[39]
Islet Transplantation	Human xenograft in immunodeficient mice	Xeno- and allogeneic islet transplant using deceased human donors and HLA-A2 transgenic mice; recipients were Treg-deficient mice.	CAR-engineered CD4^+^CD25^+^FoxP3^+^CAR-Tregs	HLA-A*02	Exhibited antigen-dependent *in vivo* suppression independent of TCR signaling.	[40]
Skin Transplantation	Mice	Allogeneic transplant using HLA-A2 transgenic C57BL/6 donor mice.	CAR-engineered CD4^+^CD25^+^FoxP3^+^CAR-Tregs	HLA-A*02	dsCAR-Tregs reduced rejection in naïve but not sensitized recipients.	[41]
Skin Transplantation	Human xenograft in NSG mice	Xenograft of tissues from HLA-A02-positive human donors into HLA-A02-negative NSG mice.	CAR-engineered CD4^+^CD25^+^FoxP3^+^CAR-Tregs	HLA-A*02	Specific, stable, efficacious, and safe in preclinical models.	[42]
Skin Transplantation	Human xenograft in NSG mice	Xenograft of HLA-A02-positive human donor tissues into HLA-A02-negative NSG recipient mice.	CAR-engineered CD4^+^CD25^+^FoxP3^+^CAR-Tregs	HLA-A*02:01	Effectively suppressed xenogeneic GvHD and reduced graft rejection.	[43]
Skin Transplantation	Human xenograft in BRG mice	Xenograft of human donor tissues into immunodeficient BRG mice.	CAR-engineered human CD4^+^ CD25^+^Treg cells specific for HLA-A2	HLA-A*02	Reduced graft damage in a human skin xenograft model compared to polyclonal Tregs.	[8]
Skin Transplantation	Human xenograft in NRG mice	Xenograft of human donor tissues into immunodeficient NRG mice.	CAR-engineered human CD4^+^ CD25^+^Treg cells specific for HLA-A2	HLA-A*02	Enhanced suppression of allospecific immune responses vs. polyclonal Tregs in human skin xenograft model.	[44]
Skin Transplantation-GvHD	Human xenograft in NSG mice	Xenograft of human donor tissues into immunodeficient NSG mice.	CAR-engineered human CD8^+^ CD45RC^low^ Treg cells specific for HLA-A2	HLA-A*02	Greater efficacy in suppressing immune responses compared to control CAR-Tregs.	[45]
Autologous Naïve Tregs	Human beings	Tregs isolated from HLA-A02-negative leukapheresis products and expanded *ex vivo*.	CAR-engineered CD4^+^CD25^+^FoxP3^+^CAR-Tregs	HLA-A*02	Feasible and safe manufacturing of high-quality naïve CAR-Tregs from ESRD patients.	[46]
Systemic Lupus Erythematosus	Humanized NSG mouse model	Engineered Tregs from human donors infused into humanized NSG mouse model of SLE.	CAR-engineered CD4^+^CD25^+^FoxP3^+^CAR-Tregs	CD19	Fox19CAR-Tregs suppressed B cells and restored clinical, histological balance.	[47]
Type 1 Diabetes	Non-obese diabetic (NOD) mice	Engineered InsB-g7 CAR-Tregs administered into autoimmune-prone NOD mice.	CAR-engineered CD4^+^CD25^+^FoxP3^+^CAR-Tregs	Insulin B chain peptide (10–23)	Prevented diabetes in NOD mice by suppressing autoimmune responses.	[48]

Summary of clinical and preclinical studies investigating the therapeutic potential of CAR-Tregs across diverse immune-mediated indications, including transplantation and autoimmunity. The STEADFAST trial represents the first-in-human, phase I/IIa clinical evaluation of TX200-TR101, a CAR-Treg therapy targeting HLA-A02, in kidney transplantation involving HLA-A02-mismatched donor–recipient pairs [18]. In solid organ transplantation, CAR-Tregs have been deployed in murine and non-human primate models of ABO-incompatible heart and islet transplantation, demonstrating antigen-specific homing, *in situ* suppression, and prolonged graft survival [38–40]. In cutaneous transplantation models, HLA-A*02-directed CAR-Tregs have been evaluated in multiple xenograft systems (NSG, NRG, BRG, and transgenic mice), where they exhibited superior graft protection and immune modulation compared to polyclonal Tregs [8, 41–45]. In autoimmune and inflammatory models, CAR-Tregs engineered to target CD19 or insulin-derived peptides effectively re-established immune tolerance in preclinical models of SLE and Type 1 diabetes. Additionally, GMP-compliant protocols for manufacturing naïve CAR-Tregs from ESRD patients demonstrated feasibility for clinical translation [46–48]. Abbreviations: BRG; BALB/c Rag2^−^^/^^−^ IL2rγ^−^^/^^−^, CD4; cluster of differentiation 4, CD25; cluster of differentiation 25, HLA-A2; Human Leukocyte Antigen A2, NRG; NOD-Rag1null IL2rgnull, NSG; NOD scid gamma, CD8; cluster of differentiation 8, CD45RC^low^; Cluster of Differentiation 45 Receptor Component low, C4d; Complement component 4d, dsCAR-Tregs; donor-specific CAR regulatory T cells. InsB-g7; Insulin B chain 10–23 peptide presented by the IAg7 MHC class II allele.

Another challenge is the inability of CAR-Tregs to suppress memory T-cell responses or prolong graft survival in sensitized recipients, despite their promising preclinical efficacy in reducing anti-HLA-A2 DSAs in naïve recipients [3, 7]. Ultimately, ∼20% of patients on the Eurotransplant waiting list are sensitized, with about 5% considered highly sensitized based on calculated panel-reactive antibody levels [19]. Prior sensitizing exposures—such as blood transfusions, pregnancies, or previous transplants—can lead to anti-HLA donor-specific antibodies (DSAs) and interferon-γ-producing memory T cells, both of which may limit the efficacy of CAR-Treg therapies [3, 7].

To address these challenges, alternative strategies such as dual CAR-Tregs for transplant rejection and relapse, and chimeric HLA antibody receptor (CHAR) T cells for sensitization, are being explored [20, 21]. Dual CARs can target two antigens simultaneously, while CHARs incorporate HLA-derived extracellular domains to direct T cells against B cells producing DSAs. CHAR constructs such as A2-CHAR and A3-CHAR are currently being investigated in preclinical studies for their selective targeting of DSA-producing B cells, which are central to antibody-mediated rejection (ABMR) [22, 23]. In parallel, combination CAR-T therapies targeting both memory B cells (Bmems) via CD19 and long-lived plasma cells (LLPCs) via B-cell maturation antigen (BCMA) have shown promise in murine transplant models and in patients with anti-HLA antibodies [24]. A clinical trial (NCT06056102) is currently underway to evaluate this dual-targeted approach in highly sensitized transplant candidates. Nevertheless, concerns remain regarding the safety of broad B-cell depletion, particularly in immunosuppressed populations. To address these multifaceted challenges, next-generation approaches such as dual CARs and bicistronic CAR-Tregs—engineered to simultaneously target alloimmune rejection and autoimmune relapse—as well as CHAR-based strategies for desensitization, may offer greater clinical utility. These approaches will require rigorous preclinical validation and thoughtfully designed clinical trials to ensure safety, efficacy, and scalability in transplant recipients with complex immunologic profiles.

### Autoimmune relapse and alloimmune sensitization

Although current clinical trials—such as STEADFAST—and preclinical studies are primarily evaluating CAR-Treg therapy for the prevention of allograft rejection in solid organ transplantation, they largely overlook the concurrent risk of relapse from pre-existing autoimmune conditions [18]. This highlights a critical and underexplored therapeutic opportunity for applying CAR-Tregs in the context of autoimmune glomerular diseases (AGDs), where targeted immune modulation must address both the induction of graft tolerance and the prevention of autoimmune recurrence. AGDs—including LN, IgAN, FSGS, and AAV—are significant contributors to ESRD and can frequently recur after RT, posing persistent challenges to long-term graft function [9–13]. Although conventional immunosuppressive regimens are effective in preventing the recurrence of AGDs, their concurrent use with CAR-Tregs can compromise the efficacy of CAR-Treg therapy, as described above [14]. LN, a severe renal manifestation of systemic lupus erythematosus (SLE), is characterized by immune complex deposition and glomerular inflammation, with recurrence rates reported in ∼35.7% of cases—rising to 48.3% in African-American patients [25]. IgAN, the most prevalent form of primary glomerulonephritis worldwide, is associated with mesangial deposition of galactose-deficient IgA1 complexes, and recurrence post-transplant is ∼28% at 11 years, influenced by biopsy protocols and duration of follow-up [26]. FSGS exhibits a challenging course post-transplant, with recurrence in ∼26% of cases; while many respond to plasmapheresis, some progress to persistent disease or graft failure [27]. In contrast, AAV tends to recur less frequently, with relapse rates ∼12%, although both renal and systemic relapses remain possible [28].

Given the distinct immunopathologic mechanisms underlying AGDs and the adverse effects associated with conventional immunosuppression, antigen-specific CAR-Treg therapy is emerging as a promising therapy. However, its application in simultaneously targeting relapse-associated antigens and donor HLA-mismatched alleles remains largely unexplored. Emerging strategies—such as bicistronic CARs, dual CARs, or combinations of two distinct CARs—are discussed further in the “Target antigen” section and may offer more precise immune modulation, potentially reducing recurrence while preserving graft integrity.

On the other hand, CAR-Tregs are inefficient at eliminating ABMR in sensitized recipients, which remains a leading cause of allograft loss and is primarily driven by DSAs targeting mismatched HLAs [3, 7]. Current therapeutic strategies, including plasmapheresis (PLEX) and intravenous immunoglobulin (IVIG), aim to reduce DSAs but are based on limited evidence and yield variable outcomes [29, 30]. B-cell–targeted therapies, such as rituximab, do not eliminate LLPCs—the major source of persistent DSA production—as these cells lack CD20 expression and are shielded in bone marrow niches [29–31]. Additional interventions such as proteasome inhibitors, complement blockers, and IL-6 pathway inhibitors provide only partial efficacy and carry the risk of broad immune suppression [32, 33]. Novel agents targeting CD38, B-cell-activating factor, and C-X-C chemokine receptor type 4 (CXCR4) are currently under investigation; however, they may unintentionally disrupt regulatory B-cell subsets [34].

Advances in CAR-T cell technologies are emerging to shape exploratory strategies for managing alloimmune sensitization in transplant recipients. CD19-directed CAR-T cells, although validated in hematologic malignancies, have shown a limited impact on DSAs, largely due to the absence of CD19 expression on LLPCs [35]. CAR-T cells targeting BCMA—which is more consistently expressed on plasma cells—have demonstrated promising results in plasma cell malignancies, yet their role in desensitization and ABMR remains to be fully defined in transplant-specific settings [36].

To address these limitations, dual-targeting CAR constructs that co-engage CD19 and BCMA have been investigated in preclinical models [24]. Such combination CAR-T cell therapy is also being evaluated in a registered clinical trial (NCT06056102) for the desensitization of highly sensitized patients awaiting RT. However, complete depletion of memory B cells and LLPCs in immunosuppressed recipients may increase vulnerability to infections, cardiovascular complications, and malignancies, emphasizing the need for more selective therapeutic strategies. An alternative and increasingly discussed platform involves chimeric autoantibody receptor (CAAR) T cells, which incorporate alloantigen or autoantigen epitopes to selectively target B cells bearing cognate B-cell receptors (BCRs), thereby sparing bystander immunity. Initial clinical data from autoimmune diseases such as pemphigus vulgaris have demonstrated high specificity and acceptable safety, although their application in transplantation remains unexplored [37]. Other potential antigen-specific constructs under early development may include CHAR-T and chimeric alloantibody receptor T (CARA-T) cells, which can employ synthetic donor HLA domains as targeting modules but may differ in their intended immunologic engagement. CHAR-T cells are being designed to sequester and neutralize circulating DSAs, with the goal of reducing plasma cell-mediated alloantibody production [21]. CARA-T cells, as a future conceptual approach, may instead engage BCRs on alloantigen-specific naïve B cells and Bmems, intervening earlier in the alloimmune cascade and offering a proactive strategy for desensitization.

### CAR and CHAR construct design

**Figure 1:**
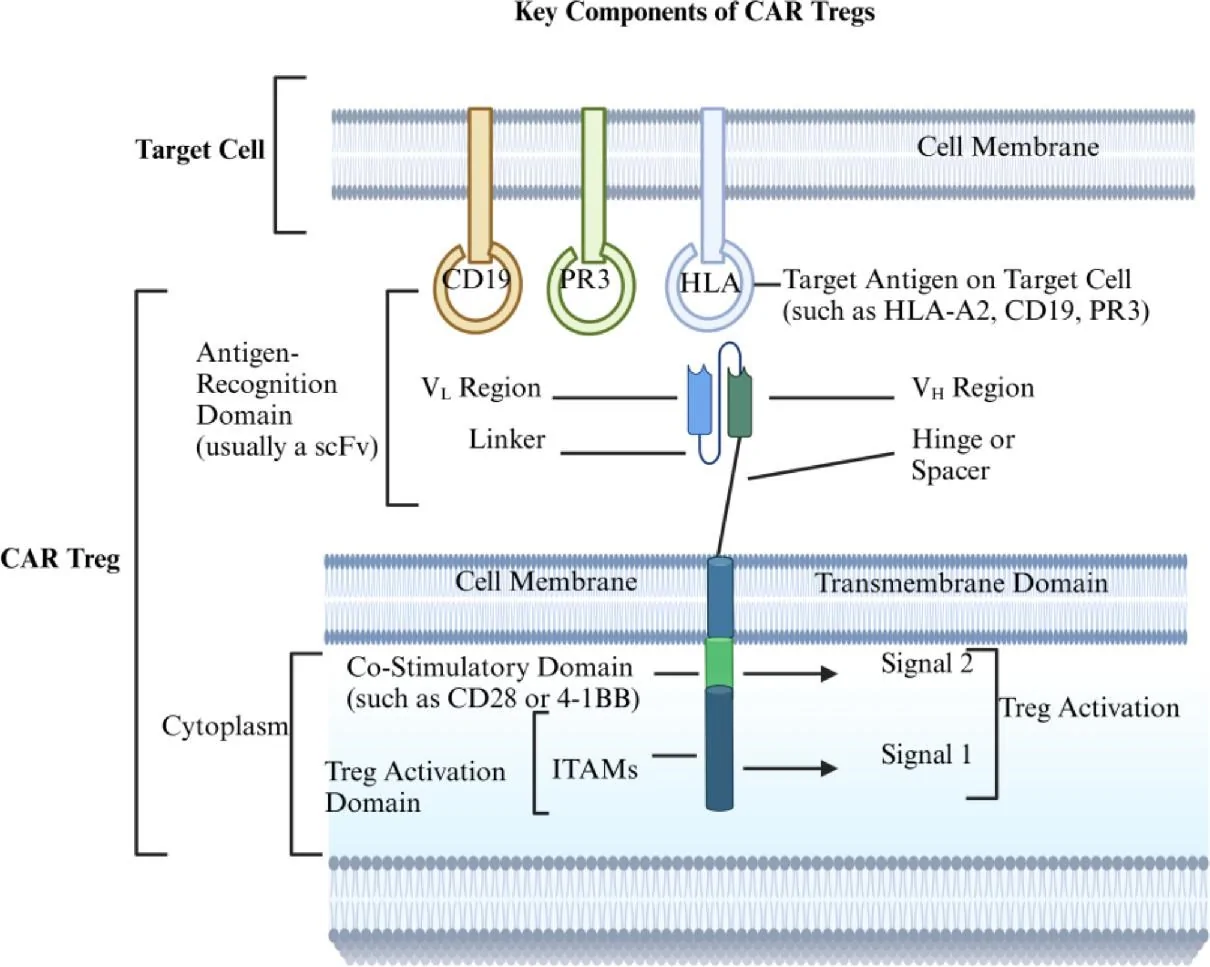
Key components of CARs. CARs consist of an extracellular antigen-recognition domain, typically a single-chain variable fragment (scFv), which provides antigen specificity. This domain can be derived from mismatched donor HLA-A2, B-cell surface markers such as CD19, or disease-specific antigens such as PR3 from ANCA-associated vasculitis. The antigen-recognition domain is linked to intracellular signaling domains via hinge and transmembrane (HTM) regions, which include a co-stimulatory domain (e.g. CD28 or 4–1BB) and a Treg activation domain, typically derived from CD3ζ. Created with BioRender.com Abbreviations: ITAM, immunoreceptor tyrosine-based activation motifs; V_H_, variable heavy chain; V_L_, variable light chain.

CARs consist of five key domains, including an antigen-recognition domain, typically a single-chain variable fragment (scFv), which enables HLA-independent antigen targeting, along with hinge, transmembrane, and cytoplasmic domains such as co-stimulatory molecules (e.g. CD28, 4–1BB) and the CD3ζ T-cell activation domain (Fig. 1) [49–51]. The antigen-recognition domain is crucial for directing CAR-expressing Tregs to the specific antigenic site, facilitating precise immune modulation. In solid organ transplantation, scFvs are typically derived from mismatched HLA alleles, preferably immunodominant mismatched ones, with minimal cross-reactivity and localized expression restricted to the graft [18, 46, 52, 53]. Various antigen binders, including scFvs, natural receptor ligands, and switchable, universal peptide-based or molecular binders, have been explored, with scFvs—derived from monoclonal antibodies—being the most widely used, enabling HLA-independent targeting while preserving the affinity and specificity of the parental antibody [54, 55].

CHARs, in contrast, retain the same modular architecture as CARs but replace the scFv with extracellular domains from specific HLA-mismatched alleles, allowing the receptor to act as a ligand for BCRs that recognize HLA epitopes. Based on the CAAR strategy, CHAR-T may be activated on BCR engagement, resulting in the release of interferon-γ, granzyme B, and perforin to selectively eliminate HLA-specific B cells [21]. This targeted approach may offer potential for pre-transplant desensitization by depleting alloantibody-producing B cells—an area where CAR-Tregs have shown limited efficacy in sensitized recipients [3, 7]. On the other hand, CARA-T cells may target BCRs on alloantigen-specific naïve B cells and Bmems, thereby preventing their activation and maturation into pathogenic subsets, and may serve as a proactive strategy for desensitization in highly sensitized transplant recipients.

The hinge (spacer) domain connects the antigen-recognition domain to the transmembrane region and plays a critical role in determining CAR surface expression, signaling potency, and effector T-cell persistence; its optimization has been shown to enhance cytotoxic function and improve therapeutic outcomes in cancer models [56, 57]. In CHARs and CARAs, which may use full-length HLA ectodomains to engage alloantibody or BCRs, the hinge may similarly influence receptor conformation and immune synapse formation, but must be tailored to accommodate the size and orientation of the HLA ligand and the unique mode of target recognition. Adjusting the hinge (spacer) domain's length and sequence modulates the engagement of CAR constructs with target cells, T-cell activation, and therapeutic efficacy. Spacer flexibility—determined by the use of rigid or flexible linkers—can alter receptor orientation, epitope accessibility, and immune synapse architecture [58–60]. Additionally, the origin of the hinge, commonly derived from CD8α or CD28, may influence receptor dimerization or interactions with endogenous proteins, thereby affecting constructs surface expression and tonic signaling [61]. CARs enable non-T cell receptor (TCR)-mediated interactions between T and target cells, with co-stimulatory domains such as CD28 or 4–1BB enhancing functionality and antitumor activity, demonstrating comparable clinical outcomes in B-cell lymphoma, including response rates and progression-free survival [50, 62, 63]. In CHARs and CARAs, similar co-stimulatory elements can be incorporated to drive T-cell responses upon recognition of donor-specific HLA antibodies or alloantigen-specific BCRs. While the signaling architecture in CHARs mirrors that of CARs, the immunological outcomes may differ, with these constructs aimed at modulating humoral alloimmunity rather than tumor-directed cytotoxicity. This difference necessitates a tailored optimization of co-stimulatory domains for specific clinical contexts.

While most CAR technology optimizations have been made in proinflammatory CD4 T cells, these findings may not fully apply to CAR-Tregs, particularly regarding co-stimulatory domains, highlighting the need for further research into their distinct roles in CAR-Treg functionality [2, 64]. CD28 and ICOS are emerging as promising co-stimulatory domains for CAR-Tregs, with CD28 enhancing their suppressive function and stability, thus supporting the maintenance of immune tolerance and regulatory phenotype [65, 66]. Rosado-Sánchez *et al.* identified ICOS as highly effective co-stimulatory domain in CAR-Tregs, enhancing their survival, function, and stability, particularly in inflammatory environments, further supporting the potential of CD28 and ICOS as optimal co-stimulatory domains for CAR-Tregs [2]. Interestingly, CD28 and 4–1BB are the most widely used co-stimulatory domains in CAR-T cells, providing a balance between initial activation and long-term persistence [5]. Dawson *et al.* evaluated second-generation CAR-Tregs incorporating 10 different co-stimulatory domains (e.g. CD28, ICOS, 4–1BB, PD-1, GITR), finding CD28 signaling to be superior in terms of functional outcomes and gene expression profiles [64]. While CD28-based CHARs may be suited for desensitization protocols, 4–1BB-based CHARs may be more advantageous for treating ABMR, where long-term immunomodulatory activity is required [21].

### CAR generations and types

**Figure 2:**
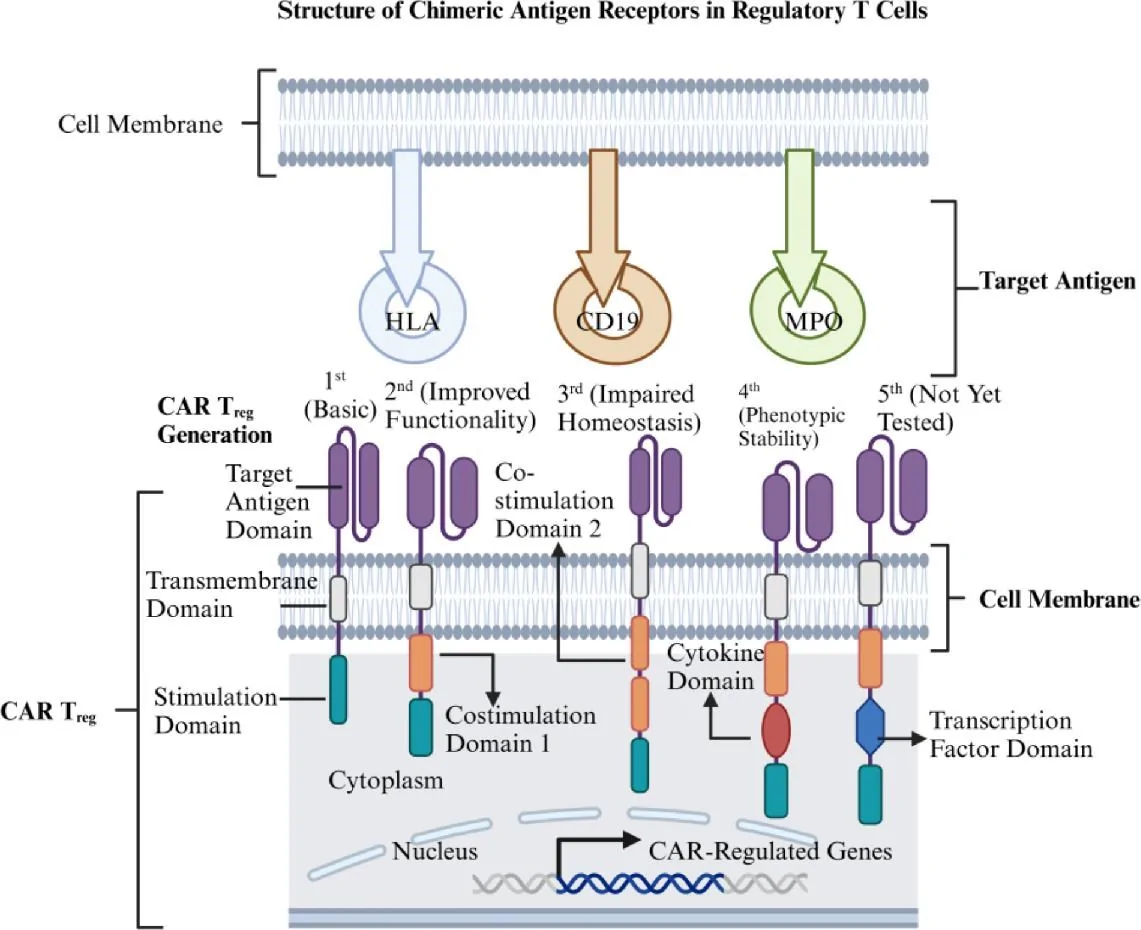
Structure of CARs in regulatory T cells. CARs facilitate non-TCR-mediated interactions between Tregs and target cells via an antigen-recognition domain and target antigen. First-generation CARs include a single stimulatory signaling domain, such as the CD3ζ ITAM-rich tail [3]. Although untested clinically, first-generation HLA-A2-CAR-Tregs were comparable to second-generation CAR-Tregs with CD28 co-stimulation in murine models with HLA-A2-mismatched skin grafts [2]. Second-generation CARs include co-stimulatory domains, such as CD28 or 4–1BB, with CD28 enhancing CAR-Treg functionality in low-co-stimulation environments [3, 50, 51]. Third-generation CARs add dual co-stimulatory domains, although benefits for T-cell homeostasis over second-generation CARs remain unsupported [38]. Fourth-generation CARs integrate transgenic cytokines or transcription factors to stabilize CAR-Treg phenotypes and enhance potency in immunocompetitive settings [53]. Fifth-generation CARs employ intracellular cytokine signaling, such as JAK-STAT kinases, but remain unexplored in CAR-Tregs [54]. Created with BioRender.com.

First-generation CARs, which incorporate only a single signaling domain—typically the CD3ζ tail rich in immunoreceptor tyrosine-based activation motifs—have not been clinically tested. However, HLA-A2-specific CAR-Tregs have demonstrated comparable efficacy to second-generation CAR-Tregs in murine models involving HLA-A2-mismatched skin grafts [2, 67] (Fig. 2). Second-generation CARs, incorporating co-stimulatory domains like CD28 or 4–1BB, enhance functionality in low-co-stimulation environments, while third-generation CARs with dual co-stimulatory domains have yet to show significant advantages for T-cell homeostasis over second-generation CARs [62, 65, 68]. Fourth-generation CARs, incorporating transgenic cytokine expression or transcription factors, aim to stabilize the CAR-Treg phenotype and enhance potency in competitive immune environments, while fifth-generation CARs with intracellular cytokine receptor signaling domains, like JAK-STAT kinases, enable a broader immune response, although their potential in CAR-Tregs remains unexplored [69, 70].

CARs can be broadly classified into six conceptual categories, each reflecting a distinct engineering approach aimed at enhancing antigen recognition, optimizing signal transduction, or improving functional durability in complex immune environments [3]. The prototypical, or monospecific, CAR consists of a scFv that mediates antigen binding, connected via a hinge-transmembrane region to an intracellular signaling domain—such as a co-stimulatory domain like CD28 or 4–1BB—and a CD3ζ chain that initiates T-cell activation [3]. In bicistronic CARs, two separate CAR or CHAR or CARA constructs can be co-expressed from a single vector, each featuring a different antigen-binding domain and intracellular signaling domain [71]. This configuration allows a single T cell to recognize and respond to two distinct antigens simultaneously, offering improved specificity. The third type is the T-cell receptor fusion construct (TRuC), which links scFvs to an endogenous TCR subunit (typically the ε-chain) with other TCR chains, followed by the armored CAR, where a T cell expresses a CAR alongside an additional protein, such as an immunologically active cytokine, to enhance CAR functionality [72, 73]. The fifth type is the CAAR, in which T cells express a CAR containing an extracellular autoantigen domain that specifically targets autoreactive B cells via their BCRs. This strategy can be adapted in the form of CHARs or CARAs to selectively engage HLA-specific B cells in the context of alloimmunity in sensitized transplant candidates [21, 74]. The sixth type can be dual in nature, accommodating either two CARs, two CHARs, or two CARAs, each incorporating different antigen-recognition domains within a single T cell, thereby enabling simultaneous but mechanistically independent engagement of two different antigens [75]. This design contrasts with bicistronic CARs, which co-express separate constructs that may function in a coordinated or sequential manner.

### Optimizing CAR-Treg therapy: subset selection and functional stability

Optimizing CAR-Treg therapy requires the careful selection of Treg subsets, efficient gene delivery, and strategies to maintain lineage stability. Human Tregs are typically isolated based on the CD4⁺CD25^high^ CD127^low^ phenotype, which includes thymus-derived Tregs (tTregs) that regulate self-tolerance, peripheral Tregs (pTregs) that respond to foreign antigens, and induced Tregs (iTregs), which are generated *in vitro* from conventional T cells under TGF-β conditions [76]. Distinguishing these subsets remains challenging due to overlapping surface markers. Epigenetically, tTregs exhibit high demethylation of the Treg-specific demethylated region, indicating stable FOXP3 expression, while pTregs and iTregs are more methylated and exhibit less stability [77, 78]. Despite their stability, tTregs may acquire memory or TH17-like phenotypes following prolonged stimulation, highlighting the importance of cytokine context during the engineering of CAR-Tregs [79, 80].

Approaches such as FOXP3 transduction have been explored to stabilize or reprogram T cells toward a suppressive phenotype. In preclinical models, FOXP3-transduced HLA-A2-CAR-Tregs maintained functionality in xenogeneic graft-versus-host disease (GvHD), while similar constructs in CD4⁺ T cells reduced inflammation in autoimmune encephalomyelitis [81–83]. In contrast, existing studies investigating CHAR constructs have predominantly utilized conventional T cells as effector platforms, without systematically evaluating how specific T-cell subsets—such as naïve, central memory, or effector memory populations—may influence *in vivo* persistence, cytolytic capacity, or immunotoxicity [22, 23]. Given the distinct functional properties and differentiation trajectories of these subsets, investigations should explore whether targeted selection could enhance the persistence, selectivity, and safety of CHAR and CARA-based strategies, particularly for pre-transplant desensitization in sensitized patients.

### Viral vectors for CAR-T cell engineering: opportunities and limitations

**Figure 3:**
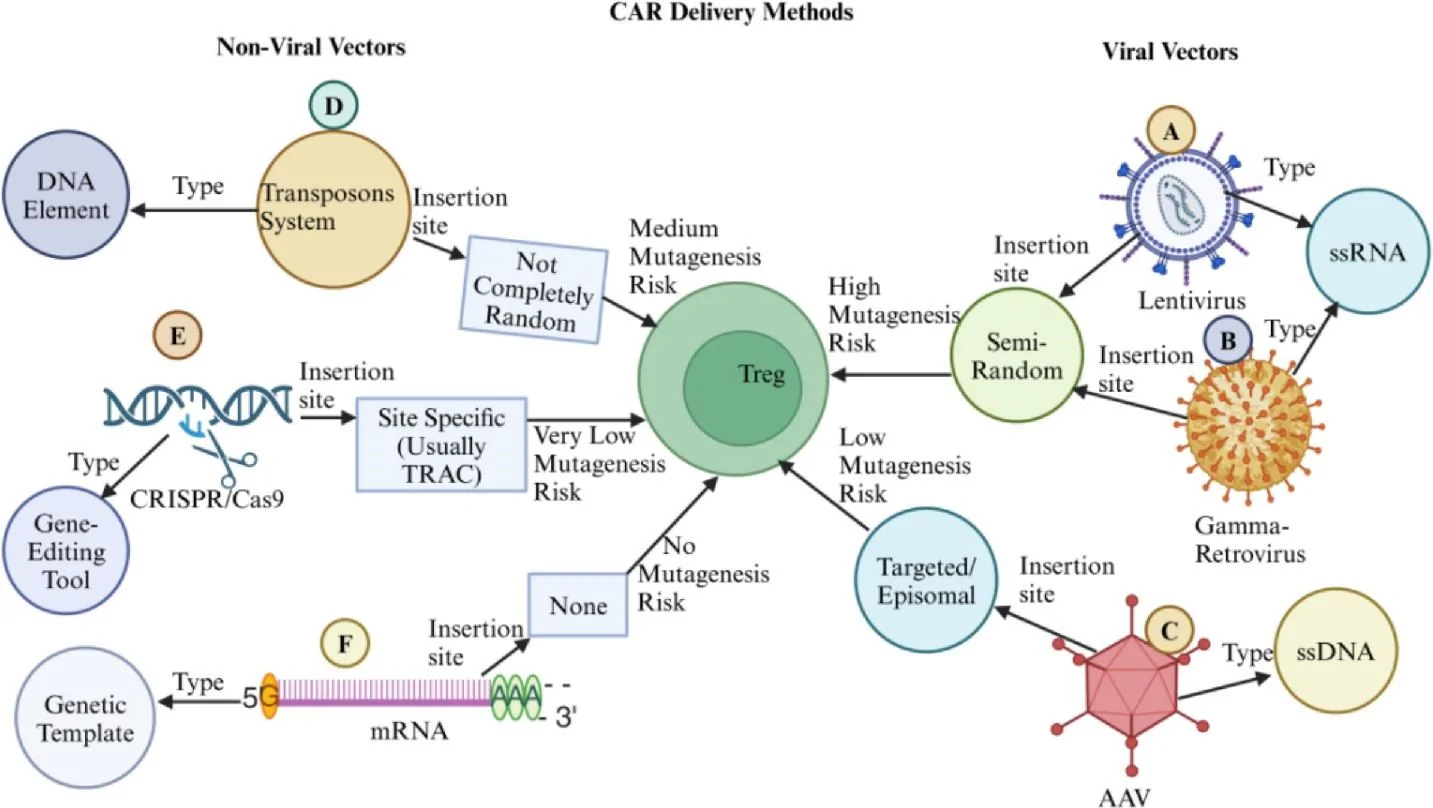
CAR delivery methods. Viral vectors include: (**A**) lentivirus, a type of ssRNA, which integrates CARs in a semi-random manner, posing a high mutagenesis risk; (**B**) gamma retrovirus, also a type of ssRNA, integrates CARs in a semi-random manner, similarly causing a high mutagenesis risk; and (**C**) adeno-associated virus, a type of ssDNA, integrates CARs in a targeted/episomal manner, resulting in a low mutagenesis risk. Non-viral vectors include: (**D**) the transposon system, a type of DNA element, which integrates CARs primarily in a targeted manner, causing a low to medium risk of mutagenesis; (**E**) CRISPR/Cas9, a gene-editing tool, integrates CARs at specific sites (usually TRAC) in a targeted manner, resulting in a very low mutagenesis risk; and (**F**) mRNA, a genetic template, does not integrate CARs and therefore poses no mutagenesis risk. Created with BioRender.com. Abbreviations: ssRNA, single-stranded ribonucleic acid; ssDNA, single-stranded deoxyribonucleic acid; CRISPR/Cas9, clustered regularly interspaced short palindromic repeats/CRISPR-associated protein 9.

Lentiviral vectors, retroviruses, and adeno-associated viruses are commonly used for CAR transduction into T cells. Lentiviruses, a subclass of retroviruses, can infect both dividing and quiescent cells, broadening the range of targetable cells for therapy [84, 85] (Fig. 3). Approximately 94% of CAR-T products are prepared using viral vectors, with >50% using lentiviruses, which are typically self-inactivating (SIN) to minimize replication risks in cell therapy [86, 87].

However, lentiviral vectors have significant limitations, including random integration that can lead to gene silencing, overexpression, or mutations, increasing the risk of adverse effects such as T-cell lymphoma and leukemia [88–91]. Additionally, lentiviruses have restricted transcriptional capacity, limiting the size of the CAR payload, particularly for dual and bicistronic constructs [92, 93]. Adeno-associated viruses may offer advantages such as low immunogenicity and a reduced risk of mutagenesis due to their episomal DNA, but their small payload capacity and potential dilution during cell expansion hinder the development of adeno-associated viruse-based dual CAR-Treg therapies [85, 94].

These limitations may also extend to emerging platforms like CHAR and CARA, which may rely on similar viral delivery systems and face challenges related to integration safety, payload capacity, and scalability. Despite these issues, alternative gene delivery methods, including transposon systems, messenger RNA (mRNA) electroporation, and CRISPR/Cas9-based strategies, may show promise for advancing CHAR-T and CAR-Treg therapies, offering safer and more cost-effective options [95–97] (Table 2).

**Table 2:** A comparison of viral and non-viral approaches for production of CAR immune cells.

Approach	Type	Production	Packing capacity	Transfection efficiency	Genome integration	Insertion site	Expression	Immunogenicity	Mutagenesis risk	Overall cost
Lentivirus	ssRNA viral vector	Labor-intensive	∼9 kb	High	Yes	Semi-Random	Stable	Low	High	Very Costly
Retrovirus	ssRNA viral vector	Moderate	∼8 kb	High	Yes	Semi-Random	Stable	Low	High	Costly
Adeno-associated virus	ssDNA viral vector	Labor-intensive	∼5 kb	High	Yes	Targeted/Episomal	Stable	Low	Low	Very Costly
Transposon	DNA Element	Simple	≈10 kb (SB)/≈220 kb (PB)	Various	Yes	Not completely random	Stable	Low	Medium to low	Cheap
CRISPR/Cas9	Gene-Editing Tool	Moderate	Moderate	High	Yes	Site-specific	Stable	Low	Very Low	Cheap
mRNA	Genetic Template	Easy	Large	Various/High	No	None	Transient	None	None	Cheap
Electroporation	Physical device	Easy	No known upper limit	High	No	None	Transient	None	None	Cheap
Biomaterials	Material science	Easy	No known upper limit	Less efficient than viral vector and electroporation	No	None	Transient	Depend on biomaterials used	None	Cheap

Information is based on a search conducted by Wu J *et al.* [109] and Chen Z *et al.* [115].

### Non-viral gene delivery strategies

Virus-free transposon systems may offer a cost-effective and efficient alternative to viral vectors, bypassing the complex processes of viral packaging, cell line expansion, and purification. Plasmid production and *in vitro* transcription (IVT) mRNA may offer even faster and more affordable production timelines [98, 99]. Transposons, like Sleeping Beauty (SB) and piggyBac (PB), can ensure stable and specific integration, preferentially targeting TA- and TTAA-rich regions, reducing the risk of disrupting neighboring genes [100]. The SB system has been particularly effective for CAR gene integration, demonstrated by the rapid generation of CD19-CAR-T cells with strong antitumor activity in xenograft models [101, 102]. The PB system, with its capacity for larger payloads (∼200 kb) and superior excision and transposition activity, can be ideal for dual and bicistronic CARs and is being explored for CAR-NK and CAR-CIK cells [96, 103–108]. Given the complexity of synthetic receptor designs, transposon systems may present a promising non-viral strategy for CHAR and CARA-T cell development, especially for constructs with large or modular payloads.

mRNA delivery for CAR therapy can be achieved through electroporation or mRNA delivery carriers, with the latter enhancing mRNA internalization and trafficking [109, 110]. While mRNA delivery carriers are limited, electroporation remains the primary method. Optimizing parameters such as electric fields and buffers enables the generation of mRNA-based CAR-T cells with selective cytotoxicity toward target cancer cells [111]. A recent study showed that inhibiting a specific DNA-sensing pathway in primary human T cells with an isotonic electroporation buffer reduced toxicity, yielding more potent CAR-T cells than viral vectors. However, electroporation is limited by significant cell death and challenges for scaled applications [109, 112]. Biomaterial-based mRNA delivery platforms, such as lipid nanoparticles (LNPs), polymeric nanoparticles, and exosomes, are being explored as alternatives, with LNPs being the most advanced [113–115]. Synthetic LNPs like C14-4 have demonstrated comparable CAR expression and cancer-killing activity to electroporation, while optimized formulations have shown effective elimination of B lymphoma [113, 116, 117]. Given the structural similarity between CAR and CHAR constructs, mRNA-based delivery could be promising for transient, integration-free expression of CHARs and CARAs.

The CRISPR/Cas9 system, a revolutionary gene-editing tool, introduces double-strand breaks in the genome through Cas9-gRNA complexes, activating DNA repair mechanisms for precise editing [118]. This technology has shown promise in CAR-T-cell engineering, as demonstrated by Huang *et al.*, who inserted an anti-CD19 CAR sequence into the PD1 gene, enhancing antitumor activity in patients with relapsed/refractory B-cell non-Hodgkin lymphoma [119]. CRISPR/Cas9 may offer advantages over traditional viral methods by enabling targeted CAR gene insertion into specific loci, such as the TCRα constant region (TRAC) in T cells, reducing mutagenesis , and improving CAR-T-cell specificity and survival [120–122]. Adeno-associated viruses are often used for homology-directed repair templates to avoid competition between CARs and TCRs [120]. However, safety concerns with CRISPR/Cas9—such as off-target mutations and immunogenicity—can be mitigated by targeting ‘safe harbor’ loci like CCR5, adeno-associated virus integration site 1, or TRAC for construct integration [118, 123–126]. CHAR and CARA constructs may also benefit from CRISPR/Cas9 for targeted integration in future applications.

### Target antigens

In autoimmune disease, CAR-Tregs can be engineered to recognize B-cell surface antigens such as CD19, CD20, CD38, and BCMA, which are variably expressed throughout B-cell development and activation [127, 128]. Targeting these molecules enables immunomodulation across critical checkpoints in the B-cell lineage, potentially attenuating aberrant humoral responses while maintaining Treg specificity. Doglio *et al.* reported that Fox19CAR-Tregs suppressed B-cell proliferation, reduced autoantibody production, delayed lymphopenia, and helped restore immune balance in a humanized SLE mouse model, without evident systemic toxicity [47]. While these findings are promising, further validation in human studies is necessary to confirm safety and efficacy. In addition to broad B-cell markers, CARs can be designed to target disease-specific antigens using scFvs derived from known autoantigens. For instance, the Smith antigen (Sm) and double-stranded DNA (dsDNA) in SLE, proteinase 3 (PR3) and myeloperoxidase (MPO) in AAV, nephrin, podocin, and CD40 in FSGS, and IgA1 or galactose-deficient IgA1 in IgAN [129].

**Figure 4:**
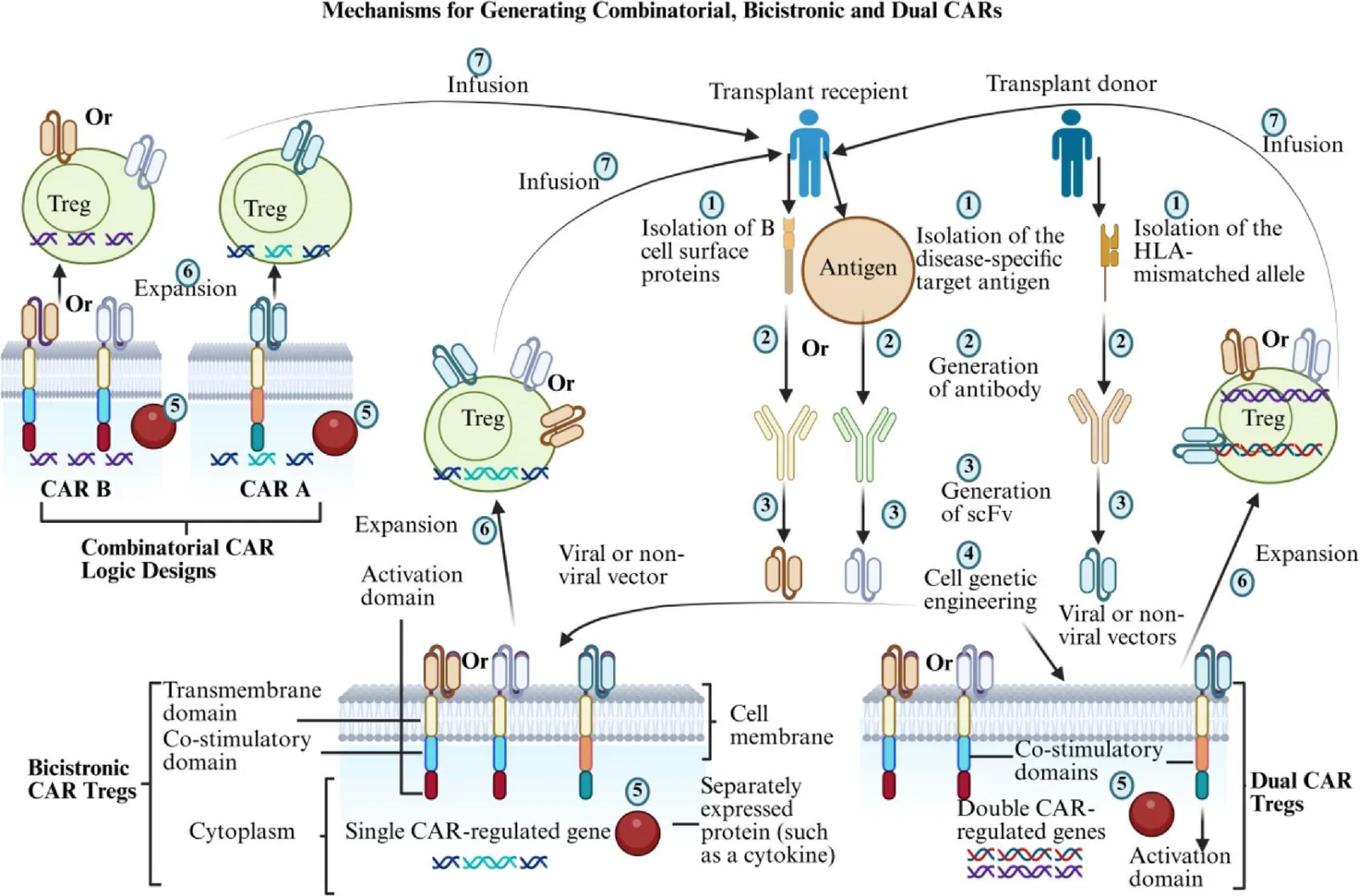
The proposed mechanism for generating combinatorial, bicistronic, and dual CAR Tregs in renal transplant involves several steps. (1) Initially, optimal Treg subtypes are isolated from the recipient (not shown here), and targeted antigens are selected, such as an HLA mismatched allele from the donor. At the same time, we can choose a target antigen from the recipient, either from B cell surface proteins such as CD19, CD20, CD38, or BCMA, or from disease-specific antigens, such as the Smith antigen in lupus nephritis or PR3 in AAV. (2) Subsequently, antibodies corresponding to the target antigens are generated, (3) followed by the creation of scFv fragments from these antibodies, and (4) genetic cell engineering is then performed using either viral or non-viral vectors. (5) To keep the engineered Tregs stable, cytokines can be administered either separately or in combination with CAR Tregs, as in fourth generation Tregs (not shown in the figure), to create a non-inflammatory environment. (6) Afterward, these CAR Tregs (either Combinatorial CAR Logic Designs, or bicistronic or dual CARs) can be expanded *ex vivo* before cryopreservation, (7) and finally, following renal transplantation, recipients can be infused with either combinatorial CAR Logic Designs or bicistronic CAR Tregs or dual CAR Tregs. Created with BioRender.com.

To address the challenge of renal allograft rejection and autoimmune disease relapse simultaneously, a dual-specific CAR-Treg strategy could be hypothetically employed (Fig. 4). One such approach may involve the use of bicistronic CAR constructs, wherein each arm of the construct is engineered to perform a distinct function [71]. The first arm can target donor-specific mismatched HLA class I or II alleles, thereby promoting immune tolerance and reducing the risk of transplant rejection. The second arm can target disease-specific autoantigens enabling suppression of autoreactive immune responses and preventing autoimmune relapse [3, 7, 128]. This dual-targeting approach is particularly relevant for patients undergoing RT for autoimmune-mediated kidney diseases such as LN, FSGS, IgAN, and AAV. In these contexts, the CAR constructs could be tailored to recognize autoantigens including dsDNA and Sm antigen in SLE, PR3 or MPO in AAV, nephrin or podocin in FSGS, and galactose-deficient IgA1 variants in IgAN.

An alternative approach may involve the use of dual CAR-Tregs, which co-express two independent CARs—one directed against mismatched HLA alleles and the other targeting disease-specific antigens or B-cell lineage markers [20, 75]. This modular design enables the simultaneous yet distinct regulation of alloimmune and autoimmune responses. Another potential approach involves combinatorial CAR logic designs, which may offer a modular configuration wherein CAR A may target donor HLA-mismatched alleles to prevent graft rejection, and CAR B can be engineered to prevent the recurrence of autoimmunity. Moreover, armored CAR-Tregs, engineered to co-express immunoregulatory cytokines (e.g. IL-10, TGF-β) or checkpoint ligands (e.g. CTLA-4), may help preserve Treg stability and function in inflammatory environments, potentially improving persistence and efficacy [3, 128]. However, the long-term impact of such modifications on Treg phenotype and *in vivo* safety require further investigation.

### Desensitization strategies in transplant candidates

In sensitized transplant candidates, alloimmune memory can result from prior exposure to donor-derived antigens via transplantation, blood transfusion, or pregnancy [3]. These antigenic targets can be broadly categorized into HLA and non-HLA alloantigens, each requiring distinct therapeutic strategies for effective desensitization and prevention of allograft rejection. In the context of HLA antigens, sensitization frequently results in the formation of DSAs against mismatched alleles, such as HLA-A* 02:01 [21]. In contrast, non-HLA alloantigens—including platelet alloantigen HPA-1a, erythrocyte antigen KEL1, and minor histocompatibility antigens such as H-Y—can elicit durable humoral immune responses, particularly in the setting of pregnancy, blood transfusion, or prior transplantation [130–132].

CHAR-T, which incorporate the extracellular domain of the sensitizing HLA molecule, allow T cells to selectively target and eliminate B cells and plasma cells that produce anti-HLA antibodies [21] (Table 3). CHAR-T cells can be particularly effective when the anticipated donor expresses the same HLA allele to which the patient has been previously sensitized. For instance, patients sensitized to HLA-A*02:01 may benefit from A2-CHAR-T cell therapy, which targets both Bmems specific to prior sensitization and B cells producing anti-A2 antibodies in response to the forthcoming graft. However, if the new graft expresses a different mismatched HLA allele—such as HLA-A03—the same A2-CHAR-T cells will target only B cells specific to HLA-A02 from prior sensitization and will not be effective against B cells reactive to the newly mismatched HLA antigen.

**Table 3:** Comparative overview of CHAR-T, CARA-T, and CAAR-T-cell platforms for targeted immune modulation.

Parameter	CHAR-T	CARA-T	CAAR-T
Target antigen	Specific HLA allele (e.g. HLA-A*02:01)	Alloantigen-specific BCR (HLA and non-HLA alloantigens)	Autoantigen or alloantigen-specific BCR epitopes
Targeted cell types	Bmems and LLPCs producing donor-specific anti-HLA antibodies	Naïve and memory B cells expressing BCRs specific to donor alloantigens	Bmems with BCRs specific to autoantigens or minor alloantigens
Binding strategy	CHAR-T expresses soluble HLA extracellular domain fused to intracellular TCR signaling motifs	Receptor mimics alloantigen structure to directly bind BCR on target B cells	Displays antigenic epitope on T-cell surface to engage pathogenic BCRs selectively
Cytotoxic mechanism	Antigen-specific granzyme/perforin-mediated killing; cytokine-mediated support (e.g. IFN-γ, IL-2)	T-cell activation on BCR engagement → cytotoxic clearance of pre-plasma B cells	Precise cytotoxicity on epitope-specific BCR recognition; low off-target lysis
Scope of application	HLA-specific desensitization in highly sensitized transplant candidates; DSA clearance	Broad desensitization to HLA and non-HLA antigens (e.g. HPA-1a, KEL1, H-Y) before DSA or plasma cell formation	Highly specific depletion of autoreactive or minor alloantigen-reactive Bmems (e.g. pemphigus vulgaris, pregnancy-sensitized minor Ags)
Clinical status	Validated *in vitro* and preclinical murine models (A2/A3 CHAR); clinical trial using dual CD19/BCMA CAR-T for desensitization (NCT06056102).	Conceptual; under development for preemptive BCR-based targeting; not yet clinically tested	Phase I in PV shows safety/efficacy; transplantation application not yet explored
Immunosuppression resistance	Retains activity under monotherapy (e.g. tacrolimus); impaired under triple regimens; FKBP12 gene editing (CRISPR) improves resistance	Unknown; likely vulnerable to calcineurin inhibitors and cytokine suppression	Likely favorable due to minimal tonic signaling and specific activation only upon cognate BCR recognition
Safety considerations	Susceptible to CRS from circulating DSAs; possible CDC/ADCC; mitigated by plasma exchange and D227K/T228A mutation to limit off-target T-cell activation	Precision targeting improves safety, but risk of broad B-cell depletion if BCR mimic is not epitope-specific	High antigen specificity demonstrated in PV; spares bystander B cells; limited risk of broad B-cell aplasia
Limitations	HLA-specific: ineffective if new graft has mismatched HLA; sensitive to anti-HLA Abs; less effective for BCR-low IgG LLPCs	Not effective post-differentiation; relies on early B-cell targeting; epitope selection is critical	Ineffective against plasma cells with downregulated BCRs (e.g. IgG LLPCs); requires validated epitope
Engineering strategies	Sequential (CHAR→CARA/CAAR) or bicistronic dual-targeting (e.g. CHAR + CARA); CRISPR-editing for IS-resistance; homing modification	Potential bicistronic or combinatorial constructs; expression-tunable dual vectors for flexible control	Sequential or combined with CAR-Tregs; suitable for tolerance protocols with spatial/temporal separation
Integration into tolerance protocols	Precedes CAR-Treg infusion; requires spatial/temporal isolation to prevent regulatory suppression	Ideal for upstream modulation before Treg tolerance programs	Can complement CAR-Tregs post-desensitization; low tonic signaling avoids interference
Monitoring readouts	Luminex SAB, C1q/C3d, MHC tetramers, cytokine profiling, single-cell RNA-seq	BCR-seq, tetramer-based B-cell tracking, scRNA-seq of memory pools	Tetramer stainings, immune monitoring, scRNA-seq for BCR specificity and lineage fate

This table presents a comparative perspective on emerging CHAR-T, CARA-T, and CAAR-T-cell platforms, outlining their conceptual designs, antigen targets, and potential roles in selective B-cell depletion for alloimmune and autoimmune settings. It summarizes the distinct recognition strategies, immunologic targets, and theoretical applications of these early-stage engineered T-cell therapies being explored for desensitization and antigen-specific immune modulation. The table provides a framework for understanding the mechanistic intent and translational rationale of CHAR-T, CARA-T, and CAAR-T approaches, which remain largely preclinical and investigational. Displayed is a side-by-side overview of CHAR-T, CARA-T, and CAAR-T constructs, focusing on their proposed target specificity, receptor engineering concepts, and potential relevance to future cell-based desensitization strategies. This comparative summary outlines the current development stage, target selectivity, and anticipated applications of CHAR-T, CARA-T, and CAAR-T cells, recognizing their promise while acknowledging their early investigational status. ADCC, antibody-dependent cellular cytotoxicity; CDC, complement-dependent cytotoxicity.

This limitation highlights the necessity of tailoring CHAR-T therapy to the specific HLA profile of both the previously sensitized antigens and the mismatched HLA alleles of the forthcoming graft [22, 23]. Similarly, CARA-T cell therapy may also offer a proactive desensitization strategy for both HLA and non-HLA alloantigens by targeting BCRs on alloantigen-specific naïve and Bmems, thereby preventing their activation and differentiation into antibody-secreting plasma cells (Table 3). In parallel, CAAR-T cells—engineered to display epitopes from relevant non-HLA antigens—may offer a complementary approach by specifically engaging and eliminating Bmems [37], thereby depleting the pool of alloantigen-specific B cells and preventing the reconstitution of pathogenic plasma cells, thus addressing a critical unmet need in current desensitization and tolerance-induction protocols (Table 3).

**Figure 5:**
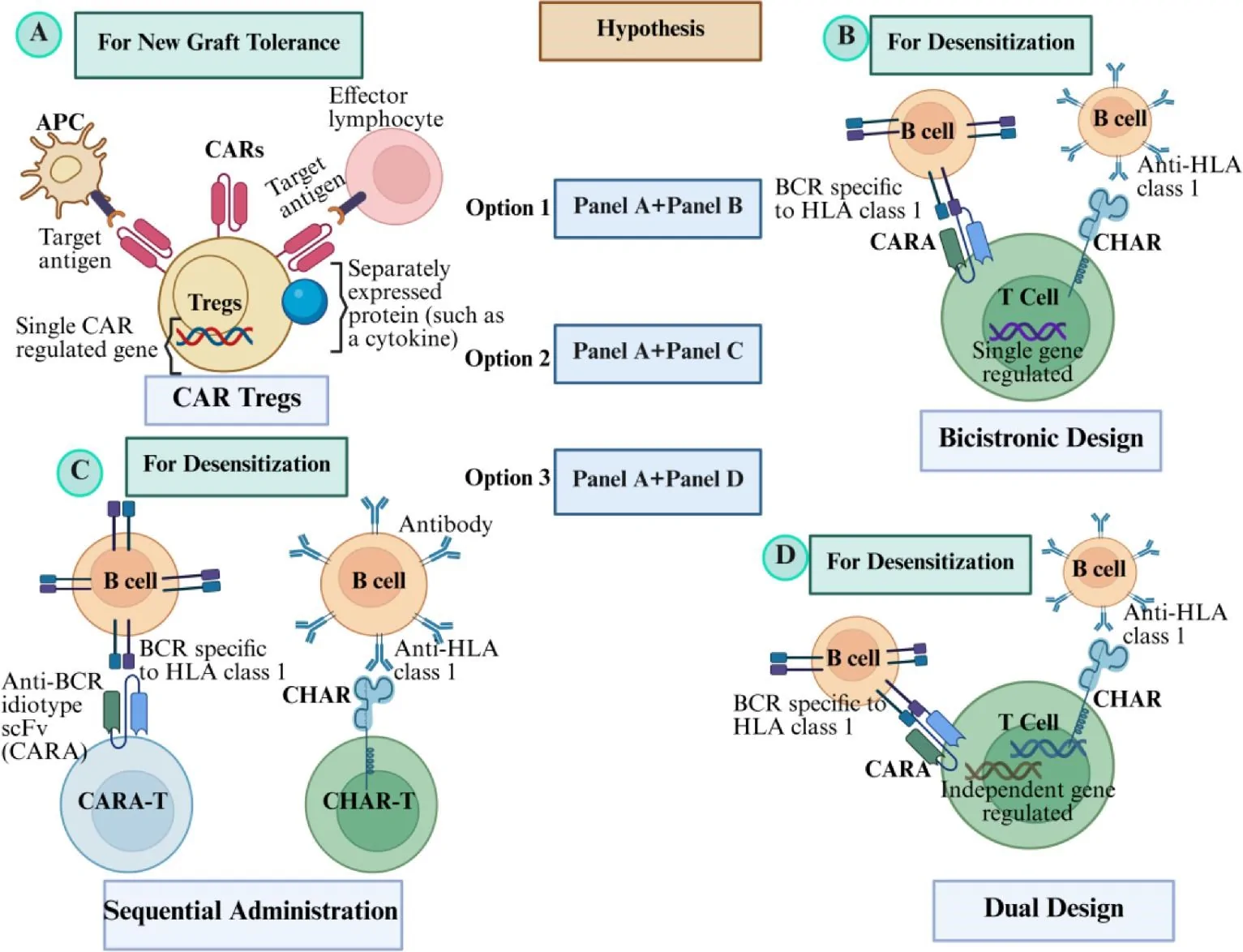
CAR-based strategies to prevent transplant rejection in sensitized recipients: (A) CAR-Tregs for tolerance: engineered Tregs expressing a CAR targeting donor antigens, with optional cytokine expression, suppress alloreactive immune responses to promote graft tolerance. (B) Bicistronic design: a single-gene-regulated CAR-T cell co-expresses CARA (targets donor-specific B cells via BCRs) and CHAR (presents HLA class I to deplete anti-HLA B cells), enabling simultaneous desensitization. (**C**) Sequential strategy: CARA-T and CHAR-T cells are administered separately to sequentially deplete donor-reactive and anti-HLA B cells, allowing staged desensitization. (**D**) Dual design: CARA and CHAR are regulated by independent gene loci, allowing controlled and tunable desensitization of different B-cell subsets. Created with BioRender.com hypothesis: combining (A) (tolerance via CAR-Tregs) with (B), (C), or (D) (desensitization strategies) could synergistically prevent graft rejection in sensitized recipients with pre-existing alloimmunity.

To address such prior sensitization, any of the strategies illustrated in Fig. 5B–D can hypothetically be applied, each specifically targeting distinct DSAs. One such strategy may involve the sequential administration of CHAR-T cells followed by CARA-T or CAAR-T cells, enabling stepwise desensitization to reduce existing DSAs and prevent activation of newly sensitized B cells. An alternative approach could involve bicistronic constructs, where a single T cell is engineered to express both CHAR and CARA (or CAAR) receptors, allowing for the potential simultaneous targeting of circulating antibodies and alloantigen-specific B cells [71]. Alternatively, dual-vector approaches can separate the two receptors into distinct expression units, providing flexibility in tuning their levels and timing to improve targeting precision [20, 75]. In parallel, to promote durable graft tolerance for new graft, CAR-Tregs-engineered to recognize donor-specific HLA mismatches, can be administered alongside or after desensitization therapy as shown in Fig. 5A. Thus, our hypothesis proposes CAR-based strategies for preventing transplant rejection in sensitized recipients by combining Fig. 5A (CAR-Tregs) with either Fig. 5B (bicistronic), Fig. 5C ( sequential), or Fig. 5D (dual integration) to induce tolerance to forthcoming graft and desensitize preformed alloimmunity resulting from prior transplantation, pregnancy, or blood transfusion (Fig. 5).

**Figure 6:**
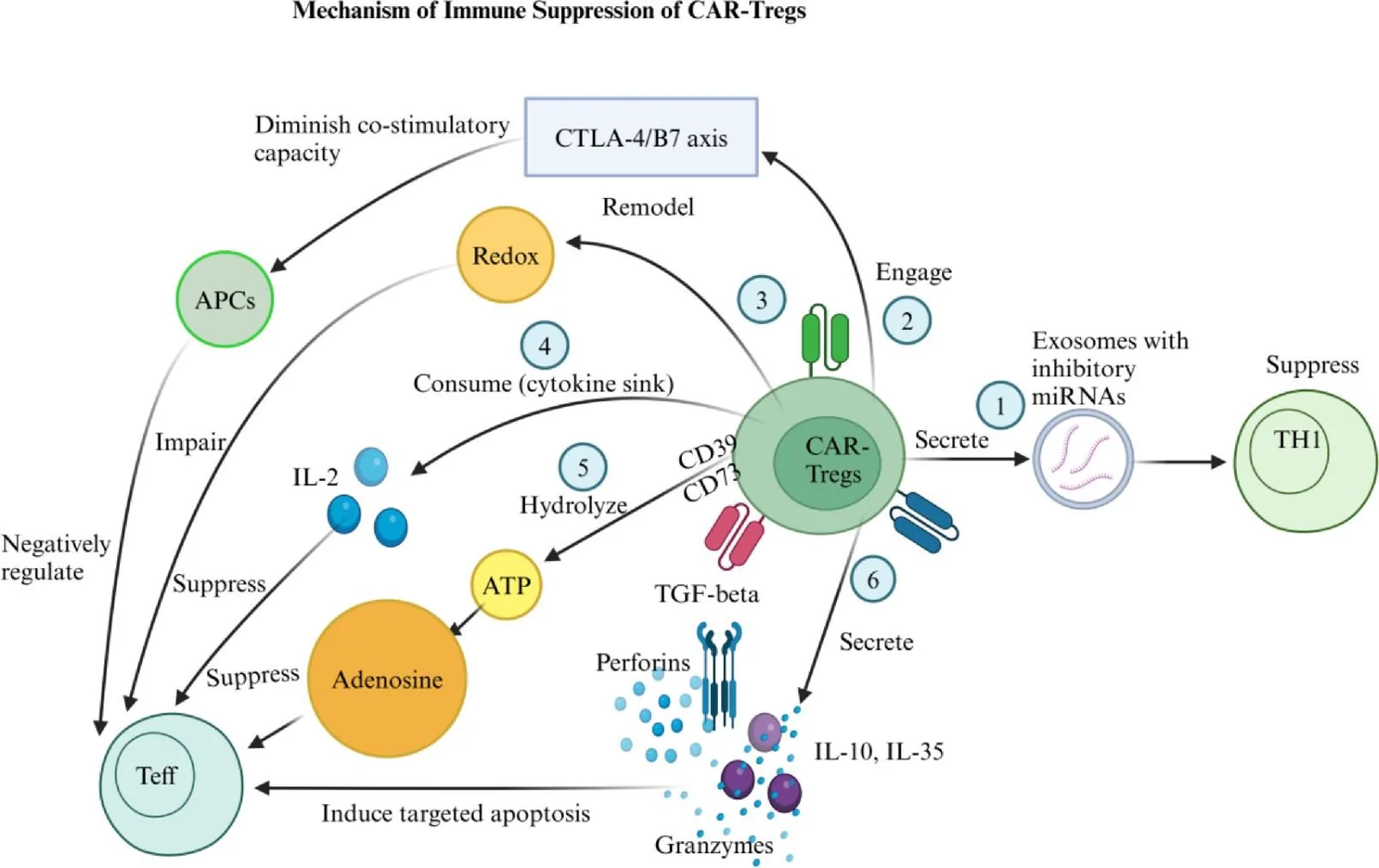
The proposed mechanism of immune suppression of Tregs in CAR Tregs is depicted. (1) CAR Tregs can secrete exosomes containing inhibitory miRNAs to suppress adaptive type 1 T helper (TH1) cell responses by inhibiting their proliferation and cytokine secretion. (2) They can engage the CTLA-4/B7 axis to reduce the costimulatory capacity of antigen-presenting cells (APCs), negatively regulating effector T (Teff) cell activation. (3) They can remodel Teff cells by reduction-oxidation (redox) to impair their activation and function, (4) consume IL-2 to suppress the growth and expansion of nearby Teff cells, (5) hydrolyze ATP via CD39/CD73 ectonucleotidase receptors to generate pericellular adenosine, downregulate Teff cells and their proliferation, and (6) secrete immunosuppressive cytokines such as IL-10, IL-35, and TGF-β to attenuate immune responses, and secrete cytolytic proteases including granzymes and perforins to induce targeted apoptosis in Teff cells. Created with BioRender.com.

CAR-Tregs function by suppressing effector T-cell responses through mechanisms such as IL-2 consumption, CTLA-4–mediated co-stimulation blockade, adenosine generation (via CD39/CD73), and secretion of immunosuppressive cytokines such as IL-10 and TGF-β as shown in Fig. 6 [1]. However, the use of CAR-Tregs alongside cytotoxic T-cell therapies requires careful consideration to avoid interference, as CAR-Tregs may suppress the activity of CHAR-T or CARA-T cells.

Strategies to mitigate this may include temporal separation, where cytotoxic therapies are administered pre-transplant and CAR-Tregs post-transplant, antigenic segregation to target distinct epitopes, and tissue-specific homing using chemokine receptor engineering to direct CAR-Tregs to the graft site. To assess the effectiveness of these cell-based therapies, high-resolution monitoring tools—such as Luminex SAB assays, flow cytometry, C1q/C3d binding assays, MHC tetramer staining, and single-cell RNA sequencing (scRNA-seq)—are essential for detailed analysis of immune responses at various stages of therapy. These tools provide insights into clonal dynamics, B- and T-cell specificity, and the persistence of engineered T cells.

#### Safety, efficacy, and functional stability of CHAR-T

CHAR-T cells, engineered to selectively recognize and eliminate B lymphocytes expressing HLA-restricted BCRs, represent an innovative strategy for targeted immunomodulation. In a pivotal study, Betriu *et al.* demonstrated that T cells transduced with an HLA-A2-directed CHAR construct effectively eradicated B-cell hybridomas bearing an HLA-A2-specific BCR, while sparing those lacking this specificity—highlighting the receptor's fine-tuned antigen discrimination [22]. Similarly, Gille *et al.* validated these findings using CHAR-T cells engineered with HLA-A2 and HLA-A3 constructs, confirming their capacity to selectively deplete B cells based on BCR specificity [23]. These results collectively demonstrate that CHAR-T cells can engage and eliminate autoreactive or antigen-specific B cells with high precision, while preserving non-targeted B-cell subsets. This selective cytotoxicity may offer a major therapeutic advantage over conventional CAR-T approaches by minimizing off-target depletion of protective humoral immunity—particularly antibodies essential for antiviral defense—thereby enhancing both safety and functional immune preservation.

Despite their antigen-selective cytotoxicity, CHAR-T cells remain susceptible to unintended activation in the presence of circulating HLA-specific antibodies. Gille *et al.* reported that exposure to anti-HLA-A2 (αHLA-A2) antibodies resulted in detectable CHAR-T cell activation, whereas no such response was elicited by anti-HLA-A3 (αHLA-A3) antibodies—suggesting differential susceptibility based on epitope structure or binding affinity [23]. This antibody-mediated stimulation can provoke cytokine secretion and may contribute to cytokine release syndrome (CRS), a potentially life-threatening complication [133]. Moreover, circulating alloantibodies may trigger complement-dependent cytotoxicity or antibody-dependent cellular cytotoxicity, thereby compromising the persistence and therapeutic potency of infused CHAR-T cells. To mitigate these immunologic threats, therapeutic plasma exchange has been proposed as a pre-conditioning strategy to deplete circulating HLA-specific antibodies prior to infusion. An additional concern involves alloreactive T cells, which may recognize and engage the extracellular domain of CHAR constructs post-infusion, thereby activating CHAR-T cells in an off-target manner. Gille *et al.* further demonstrated that this activation could be substantially—but not completely—abrogated by introducing a D227K/T228A point mutation within the CHAR scaffold [23, 134]. While this modification attenuated recognition by allo-HLA-reactive T cells, it did not fully eliminate the interaction, highlighting the need for further molecular refinements to enhance immune evasion and reduce the risk of T-cell–mediated rejection.

The effectiveness of CHAR-T cells under immunosuppressive therapy is critical in transplant settings. Preclinical studies show that CHAR-T cells retain cytotoxic function when exposed to monotherapy with agents like tacrolimus, mycophenolate mofetil, prednisone, or mTOR inhibitors. However, triple-drug regimens significantly impair their activity, primarily by suppressing key cytokines such as IFN-γ, IL-2, and TNF-α, although granzyme B release remains unaffected—suggesting granule-mediated cytotoxicity is preserved [22]. To counteract this, CRISPR/Cas9 editing targeting FKBP12 has been used to confer resistance to tacrolimus, with early results showing partial restoration of function under calcineurin inhibition, although *in vivo* validation is still required [134]. While CHAR-T cells are effective at depleting IgM-producing LLPCs, they may be less effective against IgG-producing LLPCs, which exhibit reduced BCR expression [135, 136]. This indicates that while CHAR-T cells may effectively address alloimmune B-cell responses, additional therapies, such as BCMA-CAR or anti-CD38, may be required for comprehensive plasma cell depletion in the context of desensitization protocols or ABMR treatment.

### Safety, efficacy, and functional stability of CAR-Tregs

CAR-Tregs are emerging as a promising therapeutic strategy for inducing antigen-specific immune tolerance in transplantation and autoimmune diseases (Fig. 6). Unlike conventional CAR-T cells, which are associated with toxicities such as CRS and immune effector cell-associated neurotoxicity syndrome due to proinflammatory cytokine production, CAR-Tregs are engineered to suppress immune responses and produce fewer inflammatory cytokines, potentially reducing the risk of systemic immune activation [128]. While experience from anti-CD19 CAR-T therapies in autoimmune diseases suggests a lower incidence and severity of infections compared to oncology settings, this may be attributable to less intensive prior treatments and earlier B-cell recovery [3, 7, 128]. However, clinical experience with CAR-Tregs remains limited, and the possibility of immune-related adverse effects cannot be excluded. Key challenges include off-target immune suppression, opportunistic infections, and generalized immunodeficiency, which may arise from non-specific antigen targeting or inadequate control over CAR-Treg localization and function. A major concern beyond cytotoxicity is that CAR-Tregs may suppress protective immune responses at healthy sites expressing the target antigen. This is especially relevant for widely expressed antigens like HLA-A2, where even in the absence of direct cytotoxicity, bystander suppression could impair antiviral or antitumor immunity [3]. Therefore, careful consideration of antigen tissue distribution and density thresholds is essential in CAR design. Preclinical models should assess not only cytotoxic potential but also unintended immune modulation in non-target tissues. Infection risk also remains underexplored, particularly with respect to latent viruses and opportunistic pathogens. Although CAR-Tregs produce fewer proinflammatory cytokines than conventional CAR-T cells, their potent and potentially long-lasting immunosuppressive function could dampen local immune surveillance [19, 21]. Unlike pharmacologic immunosuppressants, CAR-Tregs may accumulate or persist in specific tissues, posing additional risks for localized immune dysfunction [7]. Strategies such as combinatorial targeting, regional CAR restriction, or dose titration should be evaluated in future trials, along with the incorporation of pathogen-specific monitoring protocols [3]. Design limitations, such as poor tissue-homing ability or inappropriate co-stimulatory domain use, could lead to suppression outside the intended target tissue [3, 7]. Additionally, instability in FOXP3 expression or alterations in cytokine signaling may impair the suppressive phenotype of CAR-Tregs, reducing their therapeutic efficacy or increasing infection risks. These concerns highlight the critical need for careful antigen selection to maximize tissue specificity while minimizing systemic effects, thus ensuring both safety and effectiveness.

Gene delivery methods also have important safety implications, as lentiviruses or retroviruses integrate CAR constructs semi-randomly into the genome, raising concerns about insertional mutagenesis, which may activate proto-oncogenes or disrupt tumor suppressor genes [94]. Gene-editing technologies such as CRISPR/Cas9 may offer more targeted alternatives but can still introduce off-target edits, such as chromosomal rearrangements or genotoxicity, particularly when larger constructs or multiplex editing strategies are utilized [137–139]. Site-specific integration into safe harbor loci such as AAVS1, CCR5, TRAC, or Rosa26 may reduce these risks and improve expression stability [120].

To enhance clinical safety, suicide genes and safety switches have been incorporated into CAR-Treg constructs, such as molecules such as RQR8 or truncated EGFR (tEGFR), which may enable the conditional depletion of modified cells via monoclonal antibodies [7]. The inducible caspase-9 (iCasp9) system has also been investigated for its ability to eliminate infused cells in GvHD models. However, its activation may be affected by concurrent immunosuppressive therapies such as tacrolimus, which targets FKBP and could interfere with the dimerization mechanism required for iCasp9 function [7, 15, 16].

Another challenge is tonic signaling, which involves constitutive, antigen-independent receptor activation that can occur spontaneously and potentially lead to unwanted activation or functional dysregulation of engineered cells: a phenomenon well-characterized in effector CAR-T cells but less clearly defined in CAR-Tregs. Nonetheless, preclinical studies suggest that tonic signaling may contribute to Treg dysfunction, exhaustion, or even phenotypic instability [140]. Approaches to mitigate this may include the use of co-stimulatory domains such as 4–1BB, which may reduce basal signaling, and the exclusion of Tregs with high CAR expression prior to infusion [66, 141–143]. In addition, ablation of the endogenous TCR using CRISPR/Cas9 may reduce competition for signaling pathways and further minimize the risk of tonic signaling [121, 122].

CAR-Treg specificity has been assessed using a range of methods, including FlowPRA assays with HLA-coated beads, APCs engineered to express single HLA alleles, tetramer staining, and functional assays evaluating cytokine secretion or responder T-cell proliferation. HLA-A2–specific CAR-Tregs have demonstrated dose-dependent suppression of T-cell responses in coculture systems and effectively inhibit allospecific T-cell proliferation in mixed lymphocyte reactions using HLA-A2⁺ stimulator cells [40–45]. On recognition of alloantigen, CAR-Tregs secrete IL-10, contributing to a localized immunosuppressive environment at the graft site, an effect that may be further enhanced by constitutive IL-10 coexpression, although its efficacy *in vivo* remains context-dependent.

In NSG mouse models, bioluminescence imaging has shown that CAR-Tregs preferentially migrate to HLA-A2⁺ skin grafts, demonstrating antigen-specific homing [8, 43, 144]. In autoimmune and transplantation models, such as type 1 diabetes with HLA-A2⁺ transgenic islets, CAR-Tregs enhance graft survival, and in NRG mice bearing HLA-A2⁺ skin grafts, increased frequencies of FoxP3⁺ CD4⁺ T cells have been observed in tolerated grafts, suggesting CAR and TCR-dependent activation mechanisms that promote local immune regulation [40, 44]. While CAR-Tregs effectively suppress naïve T-cell responses and prolong graft tolerance in immunologically naïve settings, their efficacy is limited in sensitized models, where they fail to suppress IFN-γ–producing memory T cells and do not significantly improve graft outcomes [41, 145].

To address this, fourth-generation CAR-Tregs are being engineered to co-express immunoregulatory cytokines (e.g. IL-10, IL-2, TGF-β, IL-35) and molecules such as CTLA-4, which may help stabilize their phenotype and enhance suppressive potency [41, 69]. For instance, IL-10–expressing CAR-Tregs show superior suppression of effector T cells while maintaining phenotypic stability post-transduction. Parallel strategies to enhance FOXP3 expression, inhibit inflammatory signaling via dominant-negative cytokine receptors or co-inhibitory molecules, and fine-tune JAK-STAT pathway components are under investigation to support CAR-Treg survival and functionality under chronic antigen exposure [146].

To overcome resistance in sensitized or memory-rich immune environments, combination strategies are being explored, such as integrating CAR-Treg therapy with mTOR inhibitors, immunoadsorption, or tolerogenic adjuvants. The feasibility of implementing region-specific or personalized CAR-Treg therapies critically depends on aligning therapeutic constructs with population-specific HLA profiles [3, 7]. To date, HLA-targeted CAR-Treg applications have primarily been limited to living-donor–recipient transplantation, due in part to the logistical constraints of rapid HLA typing in deceased donor scenarios. However, emerging technologies such as Nanopore sequencing are showing promise for enabling real-time, high-resolution HLA typing at the point of care [147–149]. In the future, integrating HLA genotyping data into electronic health records or personal medical IDs could facilitate pre-emptive stratification and planning of individualized CAR-Treg therapies, although such infrastructure remains speculative at present [3].

To assess feasibility across diverse populations, retrospective analyses of Eurotransplant Kidney Allocation System (ETKAS) data (2017–2019) have shown that a panel of ∼12 distinct CAR specificities could theoretically address >90% of HLA-A mismatches at the antigen level [7]. This finding supports the idea that regionally or ethnically tailored CAR-Treg libraries could be developed to enhance coverage while remaining scalable. Further, scFv cross-reactivity across shared HLA epitopes might reduce the number of unique constructs needed, improving the practicality of regional implementation [6]. Multi-specific CAR-Tregs may offer additional potential by combining broader HLA targeting with lower overall cell doses, which may improve cost-effectiveness and manufacturing logistics. Current preclinical efforts have disproportionately focused on HLA-A02:01 (HLA-A2), prevalent in North America, Europe, North Africa, and West Asia (∼20% frequency in North Americans) [3, 6]. For equitable global application, future development must expand to include other commonly encountered HLA-A alleles such as HLA-A24:02, HLA-A03:01, HLA-A01:01, and HLA-A*11:01, which are more common in East Asian, South Asian, and African populations. Addressing this HLA diversity will be crucial to ensure that CAR-Treg therapies can be equitably deployed across geographic and ethnic boundaries [3, 7, 150–152] (Tables 4, 5).

**Table 4:** The most frequently occurring HLA alleles worldwide at the HLA-A, -B, -C, and -DRB1 loci.

Region	HLA-A	HLA-B	HLA-C	HLA-DRB1
North America	*02:01 22%	*35:01 7%	*04:01 15%	*07:01 9%
South and Central America	*02:12 31%	*35:43 14%	*07:02 14%	*14:02 10%
Europe	*02:01 26%	*07:02 8%	*07:01 14%	*07:01 13%
North Africa	*02:01 13%	*50:01 10%	*06:02 21%	*07:01 17%
Sub-Saharan Africa	*23:01 12%	*07:02 6%	*06:02 15%	*15:03 12%
Western Asia	*02:01 15%	*35:08 7%	*04:01 18%	*03:04 27%
North-East Asia	*24:02 23%	*51:01 8%	*01:02 17%	*09:01 11%
South-East Asia	*11:01 21%	*40:01 10%	*07:02 15%	*09:01 14%
South Asia	*11:01 13%	*40:06 12%	*06:02 12%	*07:01 18%
Australia	*34:01 38%	*13:01 24%	*04:01 25%	*14:01 13%
Oceania	*24:02 31%	*15:02 12%	*01:02 21%	*12:02 19%

Predominant allele for each HLA locus, median frequency (% as whole integer).

Information is based on a search conducted by Eskandari SK *et al.* [3] and González-Galarza FF *et al.* [6].

**Table 5:** Most common HLA alleles in North America at the HLA-A locus.

Region	*02:01	*24:02	*03:01	*01:01	*11:01
North America	22%	13%	7%	6%	5%

HLA-A allele frequency (% as whole integer).

Information is based on a search conducted by Eskandari SK *et al.* [3] and González-Galarza FF *et al.* [6].

Despite these promising advances, significant gaps remain in evaluating the manufacturing, regulatory, and economic frameworks required to enable region-specific or personalized CAR-Treg solutions at scale. These include challenges in standardizing HLA-specific construct production, streamlining approval pathways across jurisdictions, and assessing cost implications of individualized cell libraries. These areas merit focused study to unlock the broader clinical potential of personalized CAR-Treg therapy.

### Potential and challenges of CAR-Treg therapy

CAR-Treg therapies have demonstrated promising immunoregulatory effects in preclinical murine models, particularly in transplantation and autoimmune settings. However, their safety and efficacy in human transplantation remain to be fully established. A primary scientific hurdle is the absence of a universal target antigen, which increases the risk of off-target effects, generalized immunosuppression, and associated complications such as opportunistic infections and malignancies. Moreover, phenotypic instability of regulatory T cells—such as loss of FOXP3 expression and susceptibility to apoptosis via granzyme-mediated pathways following repeated antigen stimulation—poses a risk to the durability of their suppressive function [3].

The challenge of designing scFvs that avoid unintended cross-reactivity with other HLA alleles, due to shared epitope structures, further complicates antigen targeting [153]. However, this cross-reactivity could also be leveraged to design broadly reactive CARs that reduce the number of constructs needed and enhance population coverage. Optimizing construct specificity remains crucial to balancing efficacy with safety.

Alongside scientific challenges, several practical barriers continue to constrain the clinical translation of CAR-Treg therapies. A major logistical challenge is the reliance on large-scale harvesting of autologous immune cells from recipients, which involves labor-intensive processing and limits scalability while contributing to variability in product consistency and availability. To overcome this, next-generation approaches are advancing the development of universal, allogeneic CAR-Tregs derived from third-party donors or pluripotent stem cells, enabling scalable, standardized manufacturing and cryopreservation of “off-the-shelf” products [154]. These platforms could enhance accessibility, enable rigorous batch-level quality control, and allow rapid deployment in clinical settings. However, widespread implementation will depend on further progress in automated, closed-system manufacturing workflows and optimization of cryopreservation protocols to preserve cell viability and suppressive function post cryopreservation.

Regulatory challenges also remain, including the need for standardized protocols for *ex vivo* expansion, gene modification, and the establishment of reliable release criteria for identity, potency, and stability [155]. In parallel, the cost of CAR-Treg therapy—particularly in autologous formats—remains a major barrier, reflecting financial challenges similar to those encountered in CAR-T cell oncology. Realizing the full clinical potential of CAR-Tregs will require integrated solutions, including scalable manufacturing technologies, streamlined regulatory frameworks, and sustainable reimbursement models that align clinical value with economic feasibility. Robust clinical trials are critical for establishing real-world feasibility and safety. The STEADFAST trial (NCT04817774), evaluating HLA-A2-specific CAR-Tregs in renal transplant recipients, is expected to provide important insight into *in vivo* persistence and suppressive capacity [18] (Table 6). It remains uncertain whether fourth- or fifth-generation CAR-Tregs, which incorporate transgenic coexpression of stabilizing cytokines and/or transcription factors, will improve therapeutic outcomes in sensitized transplant recipients. Finally, sensitive assays to monitor CAR-Treg phenotype, persistence, and efficacy remain essential tools in guiding clinical use.

**Table 6:** Ongoing clinical trials of CAR-Tregs in solid organ transplantation, GvHD, and dual-targeted CAR-T for Highly sensitized candidates.

Trial ID and drug	Phase	Sponsor; collaborator	Official title	Target antigen	T-cell composition
NCT04817774 TX200-TR101	I/II	Sangamo Therapeutics	Multicentre Open-Label Single Ascending Dose Dose-Ranging Phase I/IIa Study to Evaluate Safety and Tolerability of an Autologous Antigen-Specific Chimeric Antigen Receptor TRegulatory Cell Therapy in Living Donor Renal Transplant Recipients.	HLA-A2	CD4^+^/CD45RA^+^/CD25^+^/CD127^low/neg^
NCT05234190 QEL-001	I/II	Quell Therapeutics Limited	A Single-arm, Open-label, Multi-center, Phase I/II Study Evaluating the Safety and Clinical Activity of QEL-001, an Autologous CAR-T Regulatory Cell Treatment Targeting HLA-A2, in HLA-A2/A28neg Patients That Have Received an HLA-A2pos Liver Transplant.	HLA-A2	CD4^+^/CD45RA^+^/CD25^+^/CD127^low/neg^
NCT05987527 TX200-TR101	I/II	Sangamo Therapeutics	Long-Term Follow-Up of Patients Who Have Received an Autologous Antigen-Specific Chimeric Antigen Receptor T Regulatory Cell Therapy (CAR- Treg Therapy, TX200-TR101) in a Prior Clinical Study.	HLA-A2	CD4^+^/CD45RA^+^/CD25^+^/CD127^low/neg^
NCT05993611 CD6-CAR-Tregs	I	City of Hope Medical Center	A First-in-Human Study to Evaluate the Safety, Feasibility and Tolerability of Allogeneic CD6 Chimeric Antigen Receptor T Regulatory Cells (CD6-CAR-Tregs) in Patients With Chronic Graft-Versus-Host Disease (cGVHD) After Allogeneic Hematopoietic Cell Transplantation (alloHCT).	CD6	CD4^+^/CD45RA^+^/CD25^+^/CD127^low/neg^
2022–002440-40, TX200-KT03 TX200-TR101	I/II	Sangamo Therapeutics France SAS	Long-Term Follow-Up of Patients who have received an Autologous Antigen-Specific Chimeric Antigen Receptor T Regulatory Cell Therapy (CAR- Treg therapy, TX200-TR101) in a prior clinical study.	HLA-A2	CD4^+^/CD45RA^+^/CD25^+^/CD127^low/neg^
NCT06056102	I	National Institute of Allergy and Infectious Diseases (NIAID)	Autologous Chimeric Antigen Receptor Engineered T Cell Immunotherapy for Desensitization in Patients Awaiting Kidney Transplantation.	BCMA and CD19	CD4^+^ and CD8^+^ T cells

### Future directions and therapeutic potential of CHAR-T

CHAR-T cell therapy are emerging as a novel immunotherapeutic platform to selectively eliminate HLA class I-specific alloreactive B cells. However, increasing clinical evidence highlights the pivotal role of de novo anti-HLA class II antibodies in mediating ABMR, correlating with increased graft loss and adverse long-term outcomes [156]. Thus, expanding CHAR-T cell strategies to target HLA class II antigens is not only a logical next step but a necessary one to comprehensively address humoral sensitization in transplant recipients.

The relatively limited polymorphism within immunodominant HLA class II loci offers an opportunity to develop a panel of CHAR constructs capable of covering a broad patient population. This could facilitate pre-transplant desensitization in highly sensitized individuals with rare donor matches. Conversely, treatment of ABMR may necessitate more individualized CHAR libraries, specifically engineered against patient-specific DSAs, given their diversity and epitope complexity. A critical limitation of current CHAR approaches lies in their inability to directly deplete LLPCs, which are central mediators of persistent alloantibody production [135, 136]. Therefore, combinatorial strategies incorporating LLPC-targeted agents or bone marrow–homing delivery platforms may warrant investigation to achieve durable alloantibody suppression. Another concern is the potential immunogenicity that could arise from exposing the antigen to which the patient is sensitized. While immunogenicity remains a theoretical issue due to antigen re-exposure via therapeutic targeting, transplant recipients are routinely exposed to HLA molecules through donor cell turnover, soluble HLA release, and HLA-bearing extracellular vesicles (e.g. exosomes). Given this ongoing exposure, CHAR-T cell therapy may not significantly increase the risk, although this hypothesis requires systematic validation [21, 157].

Ultimately, the safe and effective clinical translation of CHAR-based immunotherapy will demand a multidisciplinary, multi-center approach involving immunologists, nephrologists, hematologists, and cellular therapy experts. Rigorous preclinical modeling, followed by well-designed early-phase clinical trials, will be essential to optimize construct design, evaluate off-target effects, and assess long-term immunologic tolerance. With such integration, CHAR-T cell therapy may hold the potential to redefine desensitization protocols and improve access to transplantation for immunologically disadvantaged patients.

## CONCLUSION

CAR-Treg, CHAR-T, CARA-T, and CAAR-T therapies may hold potential based on early preclinical studies for managing alloimmunity, autoimmunity, and desensitization. However, several challenges remain, particularly in optimizing construct designs, such as the choice between combination, dual, or bicistronic approaches. For CAR-Tregs, further investigation is needed to identify optimal target antigens, improve delivery methods, and ensure stable Treg function. Similarly, CHAR-T cells must address concerns such as off-target activation and immune evasion. Future studies should focus on refining these approaches, optimizing design and delivery, and thoroughly assessing safety profiles in preclinical settings before considering clinical application.

### Ethics approval

The study was approved by the Medical Ethics Committee of Xiangya Hospital of Central South University and followed the Declaration of Helsinki.

## Data Availability

All data included in this study are available upon request from the corresponding author.
